# 

**Published:** 1845-09

**Authors:** 


### CONTENTS

OF THE

### MEDICAL EXAMINER.

NEW SERIES—NO. IX.—SEPTEMBER, 1845.

### ORIGINAL COMMUNICATIONS.

Observations on the Constitutions, according to the classification of Recamier. By Charles B. Voigt, M. D., - - - - - - 519
Two Cases of Febrile Caloricity. By Bennet Dowler, M. D., of New Orleans, - - - - - - - - - - - - 525
Solar Asphyxia, or Coup de Soleil, - - - - - - - 526
Endermic use of Tobacco. By Wm. Beck Diver, M. D., of Cincinnati, 527
Case of Dropsy of the Ovum. By John E. H. Ligget, M. D., of Frederick county, Maryland, - - - - - - - - 529

### CLINICAL LECTURES AND REPORTS.

PHILADELPHIA HOSPITAL,

*Saturday, February* 15, 1845.

Clinic of Professor Dunglison.
Chronic Gastritis, - - - - - - - - - 534
Gastrorrhœa, - - - - - - - - - - 536
Bronchorrhœa, - - - - - - - - - - 536
Rheumatism, - - - - - - - - - - 537
Pathological Specimens, - - - - - - - - 539

### BIBLIOGRAPHICAL NOTICES.

Traité d'Hygiène Publique et Privée, par Michel Levy, Médecin Ordinaire de Première Classe et Professeur d'Hygiène et de Médecine Légale à l'Hôpital Militaire de Perfectionnement de Paris, 541

First and Second Reports of the Commissioners for inquiring into the state of Large Towns and Populous Districts. Presented to both Houses of Parliament by command of her Majesty, - - - 541

Caloric; its Medicinal, Chemical, and Vital agencies on the Phenomena of Nature. By Samuel L. Metcalfe, M. D., of Transylvania University, - - - - - - - - - - - 541

The Pennsylvania Journal of Prison Discipline and Philanthropy, - 541

Elements of Chemistry, Theoretical and Practical. By George Fownes, Ph. D., Chemical Lecturer in the Middlesex Hospital School, and to the Pharmaceutical Society of Great Britain. With numerous illustrations. Edited, with additions, by Robert Bridges, M. D., Professor of General and Pharmaceutical Chemistry in the Philadelphia College of Pharmacy, &c. &c., - - - 549

### EDITORIAL.

Clinical Medicine and Surgery in Philadelphia, - - - - 550

Annual Announcements of Medical Lectures, - - - - - - 551

St. Louis Medical and Surgical Journal, - - - - - - - 552

To Correspondents, - - - - - - - - - - - 552

### RECORD OF MEDICAL SCIENCE.

Case of Congenital Malformation of the Heart. By John Bell, M. D., 553

On Ulcers of the Cornea. By W. White Cooper, Esq., F. R. C. S., Senior Surgeon to the North London Ophthalmic Institution, &c., 555

On the Microscopic Texture of Cancer, - - - - - 555

French Report on Vaccination, - - - - - - - - - 560

Camio-malacia, or Softening of the Bones of the Head, - - - 562
On the Use of Ioduret of Potassium in Syphilitic Affections, - - 562
M. Jobert de Lamballe, - - - - - - - - - - 563
Quarantine Laws. Extract from the Proceedings of the Physico-Medical Society of New Orleans, - - - - - - - 564
Hereditary Transmission of Insanity, - - - - - - - 566
On the Rapidity of the Passage of some Foreign Substances through the Kidneys, - - - - - - - - - - - 567
On the Dropsy which follows Scarlatina, - - - - - - - 567
Treatment of Chorea by the Use of Cannabis Indica, - - - - 568
Inflammation, Ulceration, and Induration of the Cervix Uteri, - - 570
Utility of Microscopic Investigations, - - - - - - - 572
Anterior Obliquity of the Uterus, - - - - - - - - 573
Case of Congenital Opacity of the Cornea. By Philip W. Maclagan, M. D., Staff Assistant Surgeon, - - - - - - - 574
On Mounting Preparations under Thin Glass. By Dr. J. W. Griffith, 575
On the Alleged Action of Sulphate of Quinine on the Spleen; and upon a New Mode of Examining this Organ, - - - - - - 575
Extra Uterine Fœtation, - - - - - - - - - - - 577
Recovery from apparent death by cold; remarkable effect of birch-tar, - 579
Treatment of Hypertrophy of the Heart, - - - - - - - 579
Enormous Hypertrophy of the Spleen, - - - - - - - 580
Antrum Tubæ of Rœderer. By Dr. Ritchie, Glasgow, - - - - 580
On the Remedial Efficacy of Ox-Gall, - - - - - - - 580
Remarks on Strychnia as a Remedy in Chorea, - - - - - - 582
On Frontal and Temporal Neuralgia, - - - - - - - - 582

